# Ovariotomy.—Removal of an Encysted Tumour of the Left Uterine Appendages

**Published:** 1846-04

**Authors:** 


Ovariotomy.—Removal of an Encysted Tumour of the Left Uterine Appendages. By George Southam, Surgeon to the Salford Royal Hospital and Dispensary, Manchester. 1845.

Mr. Southam has operated successfully in two cases of ovariotomy, and the present pamphlet contains an account of the second case, which was read at the Anniversary Meeting of the Provincial Medical and Surgical Association, and published in their Transactions.

The following is an outline of the case.

Mrs. S. æt. 38, has been married twenty years, but has never been pregnant. The abdomen began to swell eight years since, but her general health being good, and the menstrual function regular, it was attributed to corpulency. For the last twelve months the abdominal tumour has so increased in size as to embarrass respiration, and she has had pains in the left inguinal region. She has repeatedly refused her assent to be tapped. She does not know whether the swelling began on one or the other side, as she did not notice it until it appeared uniformly diffused above the pubes. She applied to Mr. Southam, being extremely desirous of having it extirpated.

"On examining the abdomen I found it nearly globular, and very prominent, distended to at least twice the size of a female at the full term of pregnancy, and elevating the cartilages of the ribs to a considerable extent; the parietes were perfectly smooth and natural in colour; fluctuation, though of a resisting kind, was very distinct in every part, and percussion elicited a dull sound. Change of position produced scarcely any alteration in its form. The uterus, as examined *per vaginam et rectum*, seemed to be of the natural size, but was situated higher in the pelvis than usual; the os was quite healthy, and on the abdomen being raised and depressed by an assistant, whilst the finger was placed in immediate contact with it, the uterus might be distinctly felt to rise and fall with an inclination to the left side. In the erect position it could not be made to bound away from the finger. There was no protrusion of the vaginal parietes, but an elastic swelling could be easily perceived, pressing on the left side of the uterus and upper part of the vagina; in other respects the pelvic cavity was not encroached upon. Though somewhat emaciated, her general health did not appear impaired; appetite good; tongue clean; pulse natural; bowels occasionally constipated, but easily acted on by medicine; catamenia regular, though in less quantity than formerly; felt pain at times in the left inguinal region, or whenever she lay on the left side, and in consequence of the pressure of the tumour upwards, she has been unable to lie on her back, or in any other position, excepting on the right side, for the last twelve months." P. 4.

These symptoms clearly indicated the existence of ovarian dropsy of the left side, and Mr. Southam's opinion was confirmed by Dr. Radford, who carefully examined the patient. The removal of the cyst was determined on. Preparatory to the operation, the patient was desired to abstain for a few days from animal food and stimulating liquors, the bowels were freely relieved by castor-oil, and the bladder emptied by the catheter. The temperature of the room was raised to 75° Fahrenheit.

Operation 24th, June, 1845. An exploratory incision, large enough to admit the finger, was made midway between the umbilicus and pubes. The clear blue shining surface of the cyst appeared at the opening, and as no adhesions appeared near the opening, Mr. S. punctured the cyst with a full-sized trocar.

"After from sixteen to eighteen pints of clear, lemon-coloured, slightly mucilaginous fluid had been evacuated, pressure on the parietes being well sustained during its escape, the canula was withdrawn, and using the index finger as a director, the opening was enlarged above and below with a probe-pointed bistoury to the extent of between six and seven inches. Having ascertained, by the hand introduced into the abdominal cavity, that there were no impediments to the extraction of the tumour, it was carefully drawn out, gentle pressure being continued on the abdomen. Finding it was attached to the uterine extremity of the left broad ligament by a short and slightly vascular pedicle, I tied it firmly with a single ligature of the strongest dentist's silk. The pedicle was now divided, and the tumour being removed, the margins of the wound were immediately approximated to prevent the ingress of air. After a brief interval, the wound was again opened to remove what blood had escaped internally, and to ascertain that the vessels of the broad ligament were firmly secured. The uterus and opposite ovary were also examined and found healthy. One end of the ligature being cut off, the other was left dependent at the lowest point of the wound, the edges of which were brought together by four interrupted sutures and straps of transparent tissue plaster. Upon these a broad pad was applied, and the whole being adjusted by a bandage, the patient was lifted into bed, within twenty-five minutes from the commencement of the operation. During the whole time she sustained herself remarkably well, apparently not suffering much pain, except whilst the exploratory incision was made, during which some little delay occurred, from the parietes being freely supplied with adipose tissue. No difficulty was experienced from the intestines protruding, as they were remarkably flaccid. There was very little hæmorrhage and no vomiting, but a feeling of faintness was superinduced by the evacuation of the fluid." P. 5.

The following day there was some pain in the course of the wound and in the left side, increased by pressure, with fever and sleeplessness, for which Mr. S. bled her to 14 ozs. which lowered the pulse and caused faintness. With this exception there was nothing untoward in the state of the patient, who gradually improved, and on the 12th of July she "returned home, a distance of three miles, and bore the journey well. The ligature came away on the forty-ninth day, after which the fistulous opening closed. The cicatrix of the entire wound does not measure more than four inches."

The treatment of the case after the operation consisted in keeping up the temperature of the room, carefully relieving any immediate symptom, such as flatus, a short cough, &c., and keeping the patient on low diet, such as barley-water, the barley-water and biscuit, and so on, to veal or chicken-broth with bread.

The cyst was single and oviform, and when filled with fluid weighed 31 pounds. The disease had begun in the broad ligament, the ovary being merely adherent to it.

In some remarks which Mr. Southam appends to the case, he insists most properly on the importance of a vaginal examination for diagnosticating these tumours of the ovary. He thinks that his mode of operating obviated the evils of both the major and minor incisions, and that the smallness of the opening would, in case of need, have enabled him to recede with but little danger to his patient. Mr. S. insists on the propriety of not performing ovariotomy until the inconveniences of the disease demand relief.

Mr. S. appears to feel strongly the delusive and false inferences which the statistics of this remedy have occasioned. "When this mechanical system of reflecting," he says, "is allowed to usurp the power of reason, or of knowledge acquired by experience and observation, or confines itself to counting facts without interpreting them, the principles founded on it will be liable to error."

We quite accord with Mr. Southam in these remarks, and we are glad to see the cajoling influence of these medical sums of addition and multiplication, plainly exposed by a man of Mr. Southam's practical good sense. If the value of remedies or modes of practice are to be exclusively tested by an appeal to statistics, in the way in which medical statistics always have been and always will be formed—we are quite sure that some of our most valuable resources must and ought to be abandoned.

In one of the concluding paragraphs, Mr. Southam remarks—

"The apparent facility of the operation may induce persons to undertake it who are not qualified to form a correct diagnosis of the cases in which it is most likely to be beneficial. Mere manual dexterity, unaccompanied by a careful consideration of the circumstances of the case, and the condition of the patient, never can lead to the establishment of ovariotomy on a firm foundation." P. 11.

